# Stimuli-Responsive Vesicles and Hydrogels Formed by a Single-Tailed Dynamic Covalent Surfactant in Aqueous Solutions

**DOI:** 10.3390/molecules29214984

**Published:** 2024-10-22

**Authors:** Chunlin Xu, Na Sun, Huaixiu Li, Xingchen Han, Ailing Zhang, Panpan Sun

**Affiliations:** 1 School of Bioscience and Technology, Shandong Second Medical University, Weifang 261053, China; xuchunlin2024@163.com (C.X.); 17753908079@163.com (H.L.); hxc18654780069@163.com (X.H.); 2 College of Pharmacy, Shandong Second Medical University, Weifang 261053, China; sunna@sdsmu.edu.cn; 3 College of Chemical Engineering and Environmental Chemistry, Weifang University, Weifang 261061, China

**Keywords:** surfactant, dynamic covalent bond, responsive vesicle, aggregation behavior

## Abstract

Controlling the hierarchical self-assembly of surfactants in aqueous solutions has drawn much attention due to their broad range of applications, from targeted drug release, preparation of smart material, to biocatalysis. However, the synthetic procedures for surfactants with stimuli-responsive hydrophobic chains are complicated, which restricts the development of surfactants. Herein, a novel single-tailed responsive surfactant, 1-methyl-3-(2-(4-((tetradecylimino) methyl) phenoxy) ethyl)-3-imidazolium bromides (C_14_PMimBr), was facilely fabricated in situ by simply mixing an aldehyde-functionalized imidazolium cation (3-(2-(4-formylphenoxy) ethyl)-1-methyl imidazolium bromide, BAMimBr) and aliphatic amine (tetradecylamine, TDA) through dynamic imine bonding. With increasing concentration, micelles, vesicles, and hydrogels were spontaneously formed by the hierarchical self-assembly of C_14_PMimBr in aqueous solutions without any additives. The morphologies of vesicles and hydrogels were characterized by cryogenic transmission electron microscopy and scanning electron microscopy. The mechanical properties and microstructure information of hydrogels were demonstrated by rheological measurement, X-ray diffraction, and density functional theory calculation. In addition, the vesicles could be disassembled and reassembled with the breakage and reformation of imine bonds by adding acid/bubbling CO_2_ and adding alkali. This work provides a simple method for constructing stimuli-responsive surfactant systems and shows great potential application in targeted drug release, drug delivery, and intelligent materials.

## 1. Introduction

Molecular self-assembly is a transition process from disordered to ordered, offering a bottom-up approach to constructing functional materials on multiple scales [1]. Rich types of amphiphiles, such as surfactants [2], peptides [3], and phospholipids [4], have been studied extensively in the field of molecular self-assembly. A special type of surfactant with a novel structure [5,6,7,8,9,10] consisting of a versatile organic cation and an organic/inorganic anion, has attracted extensive attention in recent years. Compared with the conventional surfactants, the self-assembly properties of this novel structure can be regulated efficiently and easily through reasonable design of the cation and anion structures.

Various structurally well-defined assemblies, such as spherical micelles [11], rodlike/wormlike micelles [12,13,14], unilamellar/multilamellar vesicles [15,16,17], and gel nanofiber networks [18], can be formed via the self-assembly of surfactants in aqueous solutions. Among these assemblies, the stimuli-responsive vesicles have drawn much attention due to their promising applications in targeted drug release [19], micro-reactors [20], and biotherapy [21]. The common approaches for preparing stimuli-responsive vesicles are the use of surfactants with double tails [22], the responsive cation/anion [23], and the addition of external additives [24] in aqueous solutions. For instance, Shi et al. successfully created pH/CO_2_ dual-switchable vesicles using gemini surfactants based on responsive cations (1-(3-aminopropl)-3-methylimidazolium chloride, AFIL) and sodium dodecyl benzene sulfonate (SDBS), and realized the fission and reversion of vesicles [25]. Wang et al. developed responsive vesicles in the system of [C_14_mim]X (X = [Imi]^−^, [1, 2, 4-Tri]^−^, [Ben]^−^, [Tet]^−^, and [1, 2, 3-Ben]^−^) with pH-sensitive anions, and realized the reversible structural transition between vesicles and micelles by alternating CO_2_ and N_2_ bubbling [26]. In addition, Huang et al. constructed a ternary supramolecular system, which made the reversible formation and dissociation of vesicles possible by introducing a light-responsive Azo@β-CD [27]. However, there have been few reports on the formation of stimuli-responsive vesicles by single-tailed surfactants with responsive hydrophobic chains in aqueous solutions for the sake of complicated synthetic procedures.

Dynamic covalent bonds (DCBs) [28,29], unlike traditional covalent bonds, can be cleaved and reformed reversibly under specific conditions, such as pH [30], temperature [31], and reductant–oxidant [32]. To date, an abundance of dynamic covalent bonds has been exploited to construct smart materials such as imine, acylhydrazone, disulfide, and boronic ester bonds. Combining the robustness of classic covalent bonds with the reversibility of non-covalent bonds, dynamic covalent bonds have been proven to be a powerful tool for the construction of adaptive and responsive vesicles, wormlike micelles, gels, and polymers. H. van Esch et al. reported that single-tailed [33] and double-tailed surfactants [34,35] are formed by non-surface-active precursors of bis-aldehyde and alkyl amines based on dynamic imine bonds, and found that the corresponding formed micelles, wormlike micelles, and vesicles can be switched by changing pH and temperature. In addition, the group also fabricated a series of conjugated polymers by using dynamic imine bonds and realized the controllable formation and interruption of the formed wormlike micelles [31]. Zhang et al. exploited various topological structures using dynamic covalent surfactants, such as bola-shaped [36] and H-shaped [37], and the obtained aggregates were the only responsive spherical micelles. Thus, it is of great potential to regulate the assembly and disassembly of vesicles using dynamic covalent bonds while modifying the hydrophobicity of single-tailed surfactants.

Herein, a stimuli-responsive vesicle system was formed by single-tailed surfactants with a responsive hydrophobic chain that was fabricated in situ by mixing an aldehyde-functionalized imidazolium cation (3-(2-(4-formylphenoxy) ethyl)-1-methyl imidazolium bromide, BAMimBr) and aliphatic amine (tetradecylamine, TDA) through a dynamic covalent bond. It was the first report on the spontaneous transition from micelles to vesicles and hydrogels in aqueous solutions without any additives. The self-assemblies are characterized by cryogenic transmission electron microscopy (Cryo-TEM), dynamic light scattering (DLS), scanning electron microscopy (SEM), rheology, and X-ray Diffraction (XRD). Additionally, the vesicle system can be reversed by adding alkali and acid/bubbling CO_2_.

## 2. Results

### 2.1. Characterization of Single-Tailed Dynamic Covalent Surfactant C_14_PMimBr

**Scheme 1:**
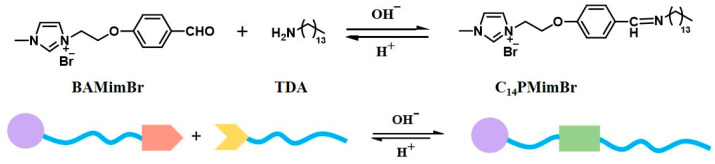
Chemical structures and schematic diagrams of the surface-inactive cationic headgroup (BAMimBr), tetradecylamine (TDA), and the single-tailed dynamic covalent surfactant, C_14_PMimBr.

**Figure 1:**
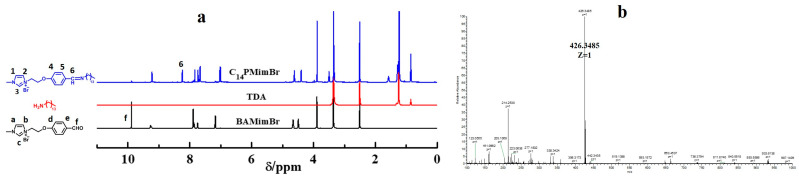
(**a**) ^1^H NMR spectra of BAMimBr, TDA, and the formed single-tailed surfactant C_14_PMimBr in DMSO-D_6_; (**b**) the MS spectrum of C_14_PMimBr.

A novel dynamic covalent single-tailed surfactant, C_14_PMimBr, was successfully designed and synthesized in situ by simply mixing TDA with the non-assembling building block BAMimBr at a molar ratio of 1:1 through dynamic covalent bonding at room temperature, as illustrated in Scheme 1. In consideration of the reactivity of dynamic covalent bonds, the pH of the solution was chosen to be 12 in this work. To confirm the formation of dynamic covalent bonds, ^1^H NMR and mass spectrometry (MS) were employed. As can be seen in Figure 1, in the ^1^H NMR spectrum of BAMimBr, a unimodal peak ‘f’ appeared at 9.91 ppm, which is the characteristic peak of aldehyde (-CHO proton) [38]. After the combination of TDA, the signal corresponding to the -CHO proton of BAMimBr nearly disappeared and a new peak ‘6’ appeared at 8.18 ppm, which is generally attributed to the -CH=N- proton [39], confirming the incorporation of BAMimBr and TDA via a dynamic imine bond, and thus, the formation of the single-tailed surfactant C_14_PMimBr was confirmed (the details of ^1^H NMR and ^13^C NMR are illustrated in Figure S2). Additionally, the appearance of the peak at 426.3485 ([M]^+^) in the ESI-MS spectrum is in accordance with the theoretical value for the C_14_PMim^+^ cation ([M]^+^ = 426.3479), indicating the formation of C_14_PMimBr (Figure S3).

### 2.2. Phase Behaviors of the Single-Tailed Dynamic Covalent Surfactant C_14_PMimBr

**Figure 2:**
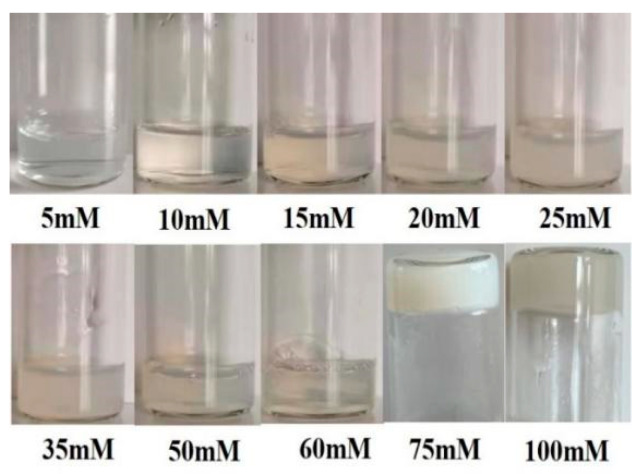
Optical photographs of C_14_PMimBr (fixing the molar ratio of BAMimBr/TDA at 1:1) solutions with different concentrations.

It is obvious that the solubility of TDA is very poor in aqueous solutions due to its long hydrophobic alkyl chain and weak hydrophilic headgroup. The building block, ionic liquid BAMimBr, was difficult to assemble into the ordered molecule aggregates because of their short alkyl chains. Therefore, the fabricated single-tailed dynamic covalent surfactant would combine the solubility of BAMimBr with the assembly ability of TDA. We systematically investigated the phase behavior of C_14_PMimBr in aqueous solutions, as shown in Figure 2 and Figure S4. Interestingly, the turbidity of the solutions increases gradually with the concentration increasing to 35 mM. From 35 to 60 mM, the turbidity of the C_14_PMimBr solutions decreases, and the assemblies at concentrations of 50 and 60 mM are unstable, which are easy to aggregate to floccules standing for two weeks. Upon increasing the concentration to 75 mM, the opalescent hydrogels begin to form. The above results illustrate that the size and morphology of assemblies would change with the increase in the concentration of C_14_PMimBr.

### 2.3. Size Distribution and Microstructure Observations of the Single-Tailed Dynamic Covalent Surfactant C_14_PMimBr

**Figure 3:**
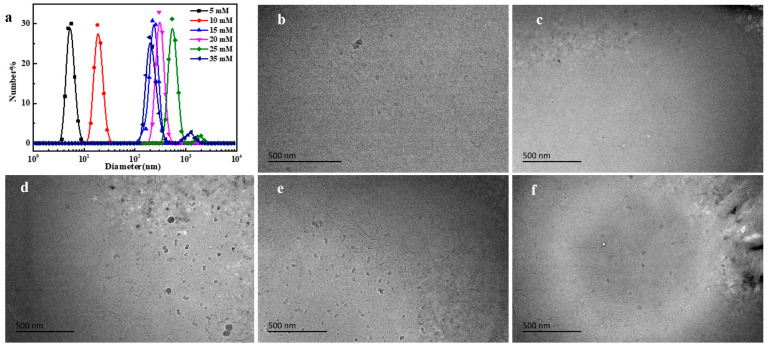
(**a**) Size distributions of C_14_PMimBr solutions with different concentrations. Cryo-TEM images of C_14_PMimBr at concentrations of (**b**) 10 mM, (**c**) 15 mM, (**d**) 20 mM, (**e**) 25 mM, and (**f**) 35 mM.

To characterize the evolution of self-assemblies with increasing concentration, two powerful tools, DLS and Cryo-TEM, were employed. The size distributions of the C_14_PMimBr aggregates at different concentrations are illustrated in Figure 3a. At a concentration of 5 mM, the hydrodynamic radii (R) of the aggregates are about several nanometers, indicating that only micelles form at this concentration [40]. Upon increasing the concentration, the size of the aggregate increases initially but then begins to decrease when the concentration reaches 25 mM. On this basis, the morphologies of the aggregate were characterized by Cryo-TEM. As shown in Figure 3b–f, the scattered vesicles were observed at a concentration of 15 mM. With increasing concentration, the population of vesicles increased. When the concentration reaches 25 mM, the formed assemblies are densely packed unilamellar and multilamellar vesicles with diameters of 100–1500 nm, as illustrated in Figure 3e, which is in accordance with the changes in aggregate size distribution. However, increasing the concentration to 35 mM gradually reduces the size distributions of vesicles, corresponding to changes in the turbidity of solutions. As reported, the slight deformation of vesicles in hydrocarbon surfactant systems can usually be found in the Cryo-TEM picture, as shown in Figure 3f, demonstrating the flexibility of vesicles [41].

### 2.4. pH—Inducing Phase Transition of Single-Tailed Dynamic Covalent Surfactant C_14_PMimBr Solution

**Figure 4:**
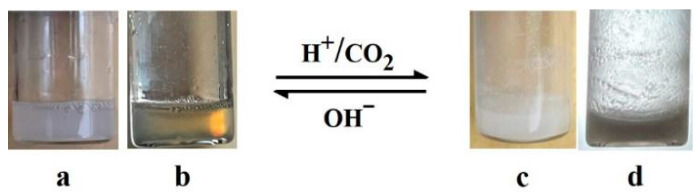
Optical photographs of the phase transition of C_14_PMimBr solutions observed without (**a**,**c**) and with (**b**,**d**) crossed polarizers at a concentration of 25 mM.

The imine bond is a typical pattern of dynamic covalent bonds that can be reversibly broken and reformed by adjusting the pH of the solutions. The effect of acidity or alkalinity on the phase transition of the C_14_PMimBr solution was explored by visual observation, with the aid of crossed polarizers. As illustrated in Figure 4, the initial state of the C_14_PMimBr solution was homogeneous opalescent under normal light (Figure 4a) and showed typical birefringent textures under crossed polarizers (Figure 4b), indicating the formation of ordered molecule aggregates [40]. The opalescent C_14_PMimBr solution was gradually precipitated into white solids (Figure 4c,d) by bubbling CO_2_ or adding acid, and this process can be reversed by adding a base, confirming that the vesicle can be disassembled and reassembled by regulating the breakage and reformation of imine bonds.

### 2.5. SEM Observation and Rheology Properties of the C_14_PMimBr Hydrogels

**Figure 5:**
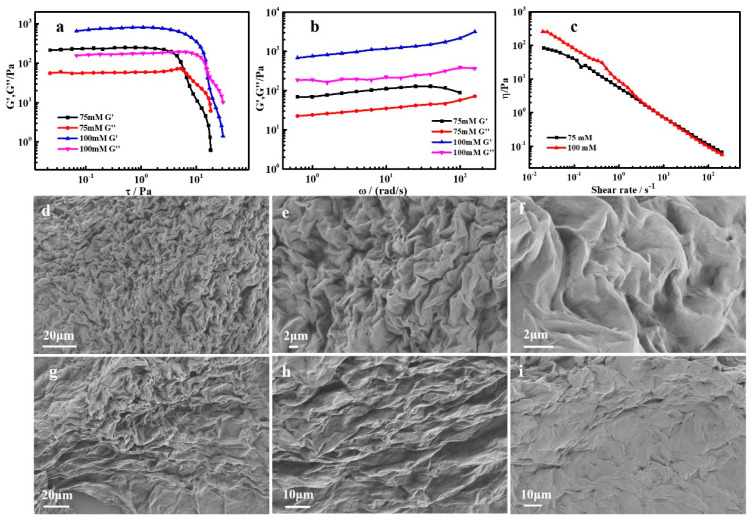
(**a**) Dynamic oscillatory strain sweep tests (from 0.1 Pa to 1000 Pa), fixing the frequency at 1 Hz; (**b**) frequency sweep tests (from 0.1 rad/s to 100 rad/s), fixing the shear rate at 1 Pa; (**c**) viscosity as a function of shear rate for the C_14_PMimBr hydrogels at concentrations of 75 mM and 100 mM. SEM photographs of the C_14_PMimBr hydrogels at concentrations of 75 mM (**d**–**f**) and 100 mM (**g**–**i**).

Rheology is a powerful method for characterizing the viscoelasticity and mechanical strength of the hydrogel system. The solid-like network structure of the hydrogels that are implanted to be sheared under increasing stress breaks suddenly at a critical shear stress, τ*, beyond which a Newtonian-like flow occurs. The τ*, namely “yield stress”, reflects the strength of the microstructure of the hydrogels. Herein, we systematically investigated the rheological behavior of the hydrogel samples at constant temperature. As can be seen from dynamic oscillatory strain sweep tests (Figure 5a), the yield stress increases with the increase in the C_14_PMimBr concentration from 75 mM to 100 mM, demonstrating enhancement in mechanical strength. In addition, we exploited the frequency sweep tests to detect the viscoelasticity of C_14_PMimBr hydrogels at a constant stress of 1.0 Pa (Figure 5b). The viscoelasticity of gels can be characterized by two dynamic moduli: the storage modulus (G′), representing the elastic properties (solid-like behavior) of hydrogels, and the loss modulus (G″), estimating the viscous properties (liquid-like behavior) of hydrogels [42]. In Figure 5b, the apparent values of storage modulus (G′) are higher than those of loss modulus (G″) in the whole frequency range of all hydrogels, presenting the typical rheological characteristic of solid-like materials. Furthermore, the storage modulus (G′) and loss modulus (G″) are slightly dependent on the frequency, reflecting that these values increase slightly with an increase in frequency. When the concentration of C_14_PMimBr ranges from 75 mM to 100 mM, the absolute magnitude of storage modulus (G′) and loss modulus (G″) increases from 100, 20 to 1000, 200, respectively, which could be ascribed to the enhancement of the mechanical strength of the hydrogels by adding C_14_PMimBr. Based on this, the steady shear tests are further employed to study the flow property of hydrogels. As illustrated in Figure 5c, the viscosity of all samples decreases gradually with increasing shear rate, corresponding to shear-thinning behavior and exhibiting the typical Newtonian-like flow, indicating that the structure of the hydrogels has been destroyed [43].

To further investigate the morphology of hydrogels, the SEM observation was selected. The obtained micrographs are shown in Figure 5. In all the cases, except for Figure 5i, wrinkle-like morphologies were observed; for Figure 5i, it was more like an aggregation of several stacked micrometer-long slices. However, the morphology observed herein deviated from the typically observed entangled fibrous network of supramolecular gels, which is usually observed in primary ammonium sulfonates [44] and guanidium sulfonate-derived gels [45].

### 2.6. XRD Investigation and Density Functional Theory (DFT) Calculation

**Figure 6:**
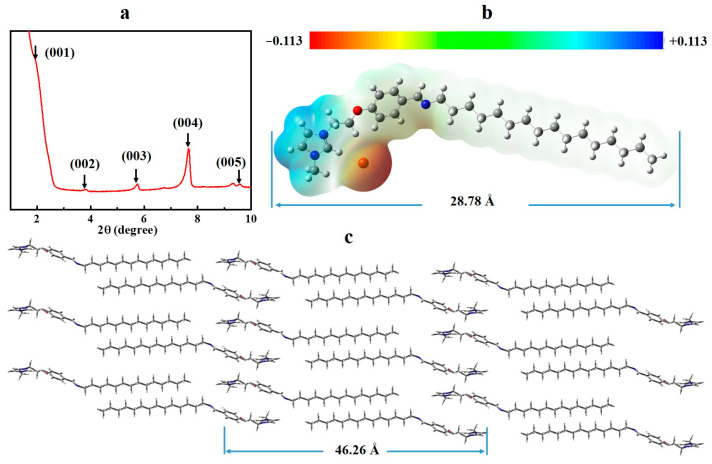
(**a**) XRD pattern of xerogel obtained by freeze-drying of C_14_PMimBr hydrogel; (**b**) optimized structures and electrostatic potentials of C_14_PMimBr by density functional theory (DFT) calculations; (**c**) schematic representation of the molecular arrangements in hydrogels.

XRD measurement is a useful approach to investigating detailed information on molecular arrangements of materials. The X-ray diffractogram obtained for C_14_PMimBr hydrogels is shown in Figure 6a. The appearance of a large number of Bragg peaks in the spectra is indicative of a highly ordered surfactant assembly [46]. The XRD pattern of the xerogel sample has five Bragg peaks (q1/q2/q3/q4/q5) with relative ratios of 1:2:3:4:5, which can be indexed 00l as the (001), (002), (003), (004) and (005), reflecting the typical feature of lamellar structure [47]. The interlayer spacing (d), consisting of the thicknesses of the amphiphile bilayer and solvent, can be calculated from the first Bragg peak (d = λ/2Sinθ). According to the first Bragg peak 2θ = 1.91, the “d” is calculated to the value of 4.62 nm. To further clarify the molecular arrangement of C_14_PMimBr in hydrogels, the density functional theory (DFT) calculation is employed. This is based on a hybrid functional B3LYP with the basis 6-31G(d, p) of the Gaussian 09 package and the obtained xyz coordinates (Figure S5 and Table S1). The fully extended length of the optimization structure of C_14_PMimBr is 28.78 Å (Figure 6a and Figure S3). It is noted that the value of “d” is more than the length of one molecule and smaller than that of two molecules, indicating chain interdigitation of C_14_PMimBr in hydrogels, as illustrated in Figure 6c [30].

### 2.7. Mechanism Analysis

**Figure 7:**
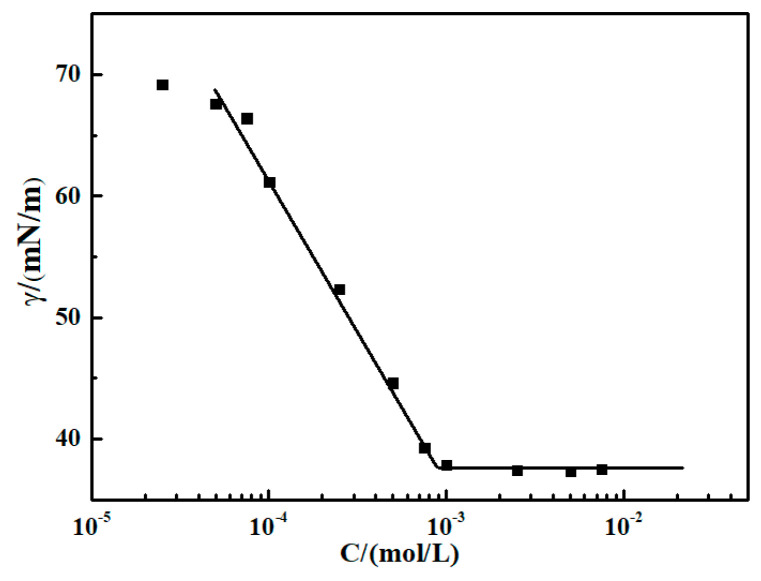
Surface tension of C_14_PMimBr aqueous solution as a function of concentration at 25 °C.

Generally, when mixing BAMimBr with TDA in aqueous solutions with pH = 12, a typical dynamic imine bond is formed, and thus, a single-tailed surfactant with a responsive chain (C_14_PMimBr) is fabricated. We systematically evaluated the aggregation behavior of surfactants in aqueous solutions by surface tension measurement. As illustrated in Figure 7, with increasing concentration, the surface tension gradually decreases until a plateau region is reached, indicating the formation of micelles. The calculated surface properties and micellization parameters of C_14_PMimBr in an aqueous solution were illustrated in Table 1. The critical micelle concentration (CMC) of C_14_PMimBr is about 89 × 10^−2^ mM, which corresponds to the concentration of the distinct break point.

**Scheme 2:**
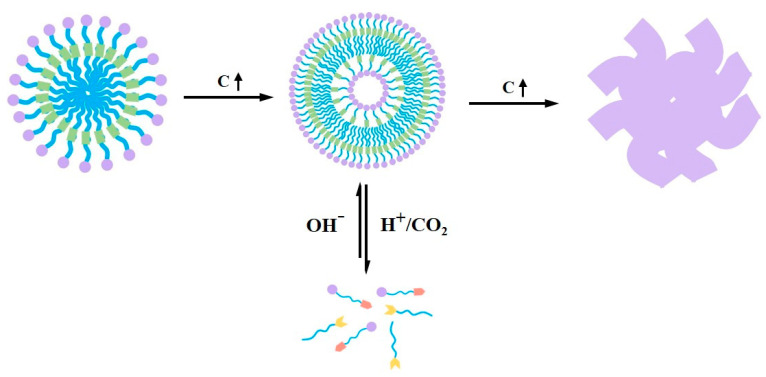
Schematic representation of the responsive behaviors of the single-tailed dynamic covalent surfactant C_14_PMimBr and its aggregates with increasing concentration and the pH/CO_2_ stimuli.

Compared to the traditional surfactants, such as alkyl trimethylammonium halide, the surfactant with an imidazolium ring headgroup possessing less electron density and the π-π interaction is apt to form vesicles [7,48]. For example, Wang et al. found that the assembly structures of the single-tailed ionic liquid (IL) surfactants, 1-alkyl-3-methylimidazolium bromides [C_n_mim]Br(*n* = 10, 12, 14), could transition from micelles to rodlike micelles and unilamellar vesicles in aqueous solutions without any additives [48]. The self-assembled structures of surfactants can be predicted by the critical packing parameter *P*, which is a classical theoretical parameter and was defined as *P* = *v*/*al* by Israelachvili et al. [49]. Here, *a* is the effective headgroup area of the surfactant molecules, and *l* and *v* are the alkyl chain length and the effective hydrophobic chain volume of surfactants, respectively. According to the values of *P*, the different structures of assemblies can be predicted, such as, spherical (0 < *P* ≤ 1/3), cylindrical (1/3 < *P* ≤ 1/2), and planar (1/2 < *P* ≤ 1) aggregates. As for C_14_PMimBr, the values of *l* and *v* were calculated using Tanford equations (as listed in SI) to be 26.8 Å and 565.4 Å^3^, respectively. Thus, the *P* value for C_14_PMimBr is 0.29, indicating that C_14_PMimBr is preferred for forming spherical micelles at relatively low concentrations. However, with increasing concentration, more Br anions bind to the imidazolium ring cations, leading to a reduction in electrostatic repulsion between head groups, which could lead to decreased *a* values and increased *P* values [48]. As a result, the change in assembly structure from micelles to vesicles and the lamellar phase is induced. Furthermore, the injection of CO_2_ or the addition of acid breaks the dynamic imine bond; thus, the single-tailed dynamic covalent surfactant is disintegrated into the non-assembling building block, BAMimBr, and the insoluble tetradecylamine. According to the above analysis, the possible mechanism of phase transition and the responsive process is proposed as displayed in Scheme 2.

## 3. Materials and Methods

### 3.1. Materials

Tetradecylamine (95%), 4-hydroxybenzaldehyde (98%), 1,2-dibromoethane (98%), N-methylimidazole (98%), CDCl_3_ (99.96%), DMSO (99.96%), and D_2_O (99.96%) were purchased from Tianjin Xiensi Biochemical Technology Co., Ltd. (Tianjin, China). Dichloromethane (CH_2_Cl_2_), acetonitrile (CH_3_CN), petroleum ether (PE), MgSO_4_, K_2_CO_3_, and KOH were obtained from Sinopharm Group Chemical reagent Co., Ltd. (Shanghai, China). All the materials were used as received without any purification. Deionized water was used in all the experiments.

### 3.2. Synthesis of 3-(2-(4-Formylphenoxy) ethyl)-1-methyl Imidazolium Bromide (BAMimBr) [50]

4-Hydroxybenzaldehyde (2 g, 16.4 mmol), 1, 2-dibromoethane (7.70 g, 41.0 mmol), and K_2_CO_3_ (6.80 g, 49.2 mmol) were dissolved in CH_3_CN (120 mL) and then the mixture was refluxed by stirring for 24 h. After the reaction, the solvent was removed under reduced pressure, and thus, the wheat solid was obtained. The crude product was purified by column chromatography (silica gel, CH_2_Cl_2_: PE = 3:2, *v*/*v*) to give compound 4-(2-bromoethoxy)benzaldehyde as a white solid. ^1^H NMR (300 MHz, CDCl_3_): δH/ppm 3.67 (t, 2H), 4.38 (t, 2H), 7.04 (d, 2H), 7.89 (d, 2H), 9.90 (s, 1H). The 4-(2-bromoethoxy)benzaldehyde was abbreviated “A”.

Compound “A” (3 g, 13.10 mmol) and N-methylimidazole (1.07 g, 13.10 mmol) were dissolved in CH_3_CN (50 mL), and the reaction was refluxed by stirring for 24 h under a nitrogen atmosphere. After the reaction, the reaction mixture was cooled to room temperature and the resulting suspension was filtered. The obtained solid was washed with diethyl ether (3 × 50 mL) and dried to give a white solid product (BAMimBr). ^1^H NMR (300 MHz, D_2_O) (Figure S1): δH/ppm: 3.77 (s, 3H), 4.42 (t, 2H), 4.56 (t, 2H), 7.02 (d, 2H), 7.32 (s, 1H), 7.48 (s, 1H), 7.78 (d, 2H), 9.65 (s, 1H). The obtained spectrum is illustrated in Figure S2.

### 3.3. Sample Preparation

The details of the fabrication process for 1-methyl-3-(2-(4-((tetradecylimino) methyl) phenoxy) ethyl)-3-imidazolium bromides (abbreviation as, C_14_PMimBr) are as follows: 0.0311 g BAMimBr and 0.0213 g TDA were mixed in 10 mL water, then the water was adjusted to pH = 12 by adding concentrated sodium hydroxide solution (10 mol/L). After aging for 12 h, the solution was used in further experiments. ^1^ HNMR (300 MHz, D_2_O): δH/ppm: 0.85 (t, 3H), 1.06 (m, 2H), 1.23 (m, 22H), 1.571 (t, 2H), 3.44 (t, 2H), 3.89 (s, 3H), 4.40 (t, 2H), 4.62 (t, 2H), 7.02 (d, 2H), 7.66–7.68 (d, 2H), 7.74 (d, 1H), 7.84 (d, 1H), 8.24 (s, 1H), 9.23 (s, 1H). ^13^C NMR (300 MHz, D_2_O): δ 161.7, 160.8, 137.0, 129.8, 128.0, 123.0, 122.8, 114.5, 68.1, 61.8, 56.2, 37.1, 31.9, 31.6, 29.6, 29.3, 27.2, 22.7, 14.1. These spectra are listed in Supplementary Materials.

The vesicles were prepared by simply mixing BAMimBr and TDA; the details of the preparation process are as follows: A total of 0.0078 g of BAMimBr and 0.0053 g of TDA were added to a 1 mL aqueous solution. The pH was adjusted to 12 by adding concentrated sodium hydroxide solution with a concentration of 10 mol/L. The mixed solution was stirred for 30 min under ultrasound (KQ-520DB (10 L), Gongyi Yuhua Instrument Co., Ltd., Gongyi, China) at a frequency of 40 KHz and a temperature of 25 °C. Hydrogels were obtained with increasing concentrations of BAMimBr and TDA, which is similar to the preparation process of vesicles. All systems were aged for at least four weeks before characterization.

To regulate the breakage and reformation of dynamic covalent bonds, concentrated hydrochloric acid and sodium hydroxide solutions were added to adjust the pH values of the samples. In the bubbling CO_2_ test, CO_2_ was bubbled into mixed BAMimBr and TDA solutions with a fixed flow of 0.1 L/min until the solutions turned turbid.

### 3.4. Characterization

Dynamic light scattering (DLS): The size distributions of the vesicles were detected by DLS using a Nanotrac Particle Size Analyzer (Nanotrac NPA 250) (Hann, Germany) and the microtrac FLEX application software program. All measurements were carried out using a laser diode (780 nm wavelength, 3 mW nominal, Class IIIB at the scattering angle of 180). The temperature was controlled using a thermostat (F31C, Julabo) with an accuracy of 25 ± 0.1 °C.

Cryogenic transmission electron microscopy (Cryo-TEM) observations: The samples were prepared in a controlled environment vitrification system (CEVS) at 25 °C. A micropipette was used to load 5 μL solutions onto a TEM copper grid. Then, the solution was blotted with two pieces of filter paper, resulting in the formation of thin films suspended on the mesh holes. After waiting for about 10 s, the samples were quickly plunged into a reservoir of liquid ethane, which was cooled in nitrogen at −165 °C. The vitrified samples were then stored in liquid nitrogen until they were transferred to a cryogenic sample holder (Gatan 626) (Pleasanton, CA, USA) and examined under a JEOL JEM-1400 transmission electron microscope (120 kV) (Tokyo, Japan) at about −174 °C. The images were recorded on a GatanMultiscan CCD and processed using a Digital Micrograph.

^1^H NMR measurements: ^1^H NMR spectra were generated on a Bruker Advance 300 spectrometer (Billerica, MA, USA) equipped with a pulse field gradient module (*Z*-axis) using a 5 mm BBO probe. The instrument was operated at a frequency of 300.13 MHz at 25 ± 0.1 °C.

Rheological measurements: The rheological measurements of hydrogels were performed on an Anton-Paar Rheometer (Graz, Austria) using a cone-plate system (C35/1° Ti L07116, diameter: 35 mm, cone angle: 1°). All measurements were carried out at 25.0 °C using a cyclic water bath.

Scanning electron microscopy (SEM): A small volume of hydrogel sample was placed on a silica wafer, and most of the colloid gel was removed using small forceps to form a thin film. The wafers were freeze-dried in a vacuum extractor for 24 h using a freeze dryer. Then, the silica wafers were subjected to gold plating and observed on a JEOL JSM-6700F FE-SEM (Tokyo, Japan).

X-ray diffraction (XRD): XRD patterns of the powder of the freeze-dried hydrogels were recorded in 2θ mode (1° min^−1^) using a DMAX-2500PC diffractometer with a Cu Ka radiation source (λ = 0.15418 nm) and a graphite monochromator.

Surface tension measurements: The surface tension measurements were carried out on a Model JYW-200B tensiometer (Chengde Dahua Instrument Co., Ltd., Hangzhou, China, accuracy ±0.1 mN/m) using the ring method. Temperature was controlled at 25 ± 0.1 °C using a thermostatic bath. Each sample was equilibrated for 10 min under the test temperature to reach equilibrium before the measurement.

Density functional theory (DFT) calculation: DFT calculation was based on hybrid functional B3LYP with the basis 6-31G(d,p) of the Gaussian 09 package.

## 4. Conclusions

In summary, a novel single-tailed dynamic covalent surfactant (C_14_PMimBr) is reported for the first time. It was fabricated in situ by simply mixing the non-assembling building block BAMimBr with insoluble tetradecylamine in aqueous solutions at pH = 12. Interestingly, the distinctive molecular structure endows rich self-assembly behaviors of C_14_PMimBr in aqueous solutions. The aggregate structures can be transformed from micelles to unilamellar and multilamellar vesicles and then to lamellar hydrogels upon an increase in C_14_PMimBr concentrations, without any additives. The formation of various vesicles was confirmed by Cryo-TEM and the molecule arrangement of C_14_PMimBr in hydrogels is deduced by the combination of XRD and DFT calculations. Interestingly, the dynamic covalent bond (imine bond) endows that the vesicles can be disassembled by bubbling CO_2_/adding acid and reformed by adding alkali. We believe that our research would broaden the horizons for constructing novel and stimuli-responsive surfactant systems and lay the foundation for applications in drug delivery, targeted drug release, and biochemical engineering.

## Figures and Tables

**Table 1:** Surface properties and micellization parameters of C_14_PMimBr in an aqueous solution at 25 °C.

Surfactant	CMC (mM)	γ_CMC_ (mN/m)	Γmax (µmol/m^2^)	Amin (Å^2^)
C_14_PMimBr	0.89	37.3	2.27	73.2

## Data Availability

Data are contained within the article and Supplementary Materials.

## Supplementary Materials

The following supporting information can be downloaded at: https://www.mdpi.com/article/10.3390/molecules29214984/s1, Figure S1: ^1^H NMR spectrum of 3-(2-(4-formylphenoxy) ethyl)-1-methyl imidazolium bromide (BAMimBr); Figure S2: ^1^H NMR and ^13^C NMR spectra of C_14_PMimBr; Figure S3: Mass spectrum of C_14_PMimBr; Figure S4: Optical photographs of C_14_PMimBr (fixing molar ratio of BAMimBr/TDA at 1:1) solutions with different concentrations; Figure S5: Geometries of C_14_PMimBr molecules optimized using the polarizable continuum model at the B3LYP/6-31G(d,p) level; Table S1: DFT calculation information for C_14_PMimBr molecule. References [51,52] are cited in the supplementary materials.
