# Supplementary material for: Stimuli-Responsive Vesicles and Hydrogels Formed by a Single-Tailed Dynamic Covalent Surfactant in Aqueous Solutions
Source: Molecules. 2024 Oct 22;29(21):4984. doi: 10.3390/molecules29214984 (PMC11547677; doi:10.3390/molecules29214984)
Supplement: Supplementary file 1 [file molecules-29-04984-s001.zip › molecules-3242274-supplementary.pdf]

Stimuli-Responsive Vesicles and Hydrogels Formed by a Single-Tailed Dynamic Covalent Surfactant in Aqueous Solutions

Chunlin Xu [1,†], Na Sun [2,†], Huaixiu Li [1], Xingchen Han [1], Ailing Zhang [3,*] and Panpan Sun [1,*]

[1] School of Bioscience and Technology, Shandong Second Medical University, Weifang 261053, China; xuchunlin2024@163.com (C.X.); 17753908079@163.com (H.L.); hxc18654780069@163.com (X.H.)
[2] College of Pharmacy, Shandong Second Medical University, Weifang 261053, China; sunna@sdsmu.edu.cn
[3] College of Chemical Engineering and Environmental Chemistry, Weifang University, Weifang 261061, China
* Correspondence: zhangal89@163.com (A.Z.); sunpanpan@sdsmu.edu.cn (P.S.)
† These authors contributed equally to this work.

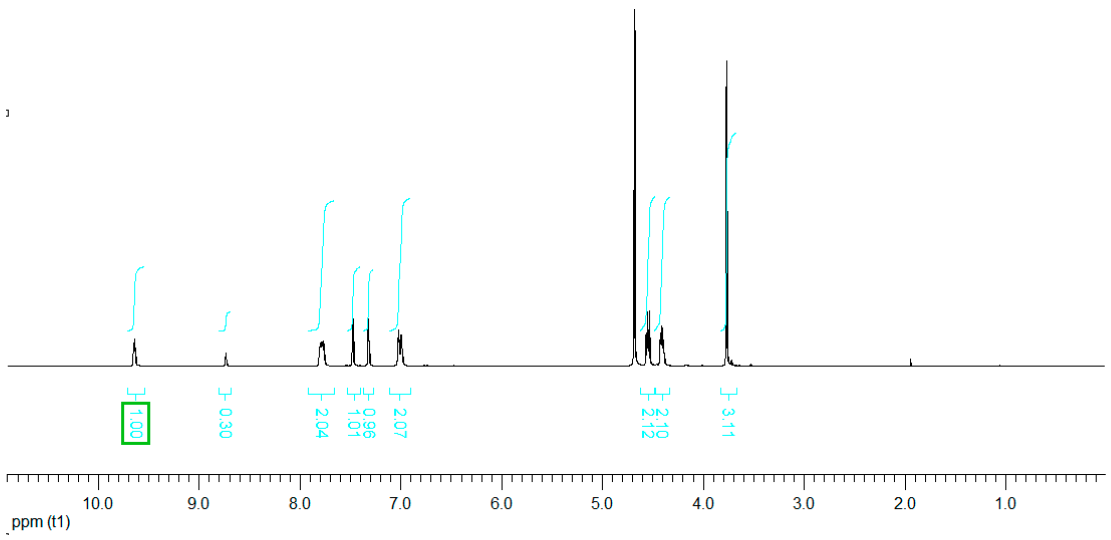

Figure S1. The ^{1}H NMR spectrum of 3-(2-(4-formylphenoxy) ethyl)-1-methyl imidazolium bromide (BAMimBr).

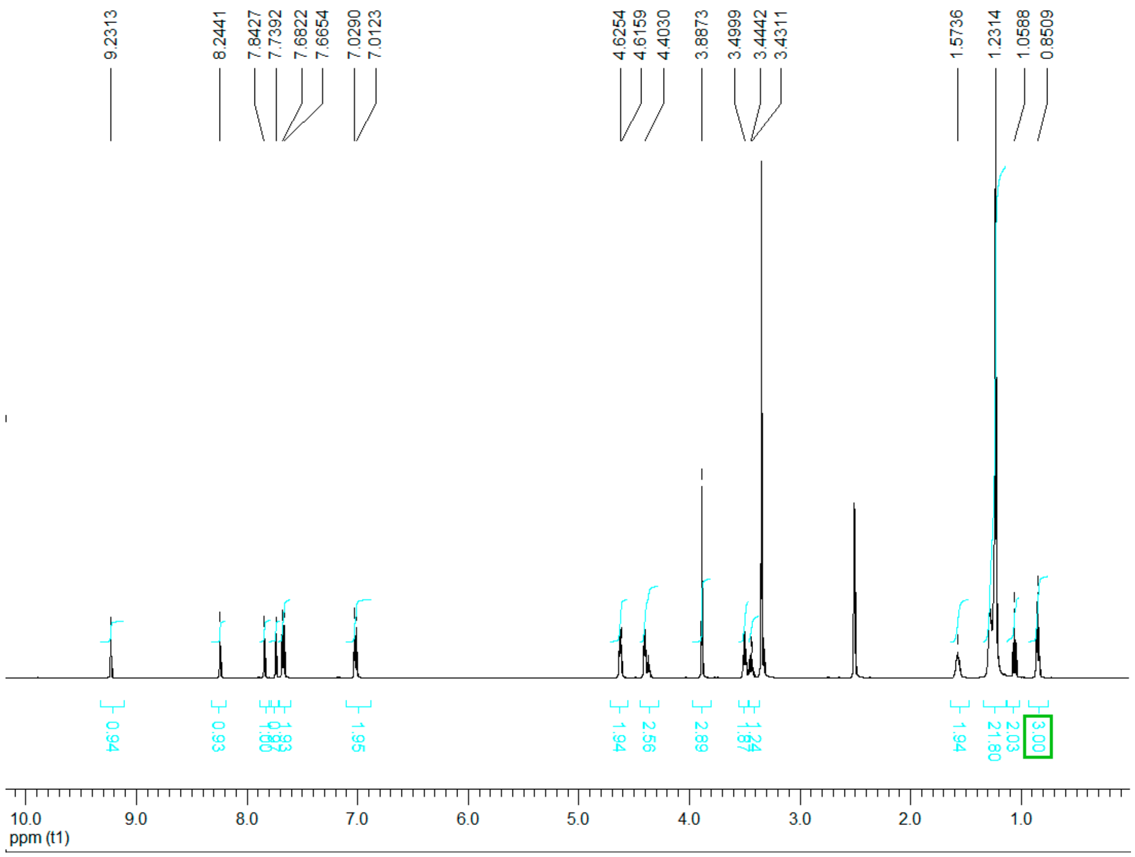

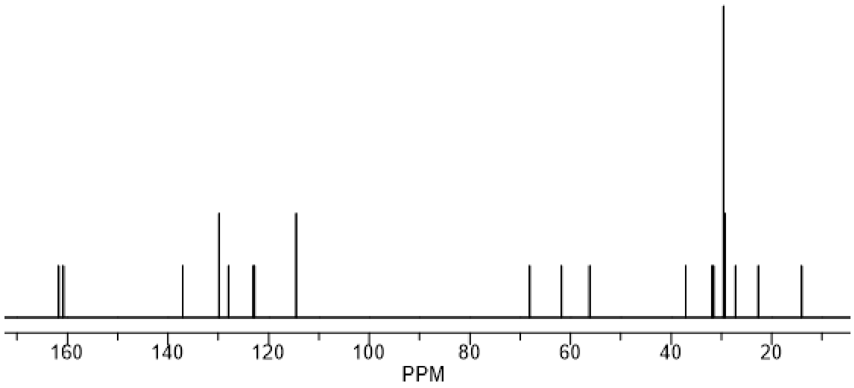

^{13}C NMR (300 MHz, D_2O) C_{14}PMimBr : δ 161.7, 160.8, 137.0, 129.8, 128.0, 123.0, 122.8, 114.5, 68.1, 61.8, 56.2, 37.1, 31.9, 31.6, 29.6, 29.3, 27.2, 22.7, 14.1.

Figure S2. The ^{1}H NMR and ^{13}C NMR spectrum of C_{14}PMimBr.

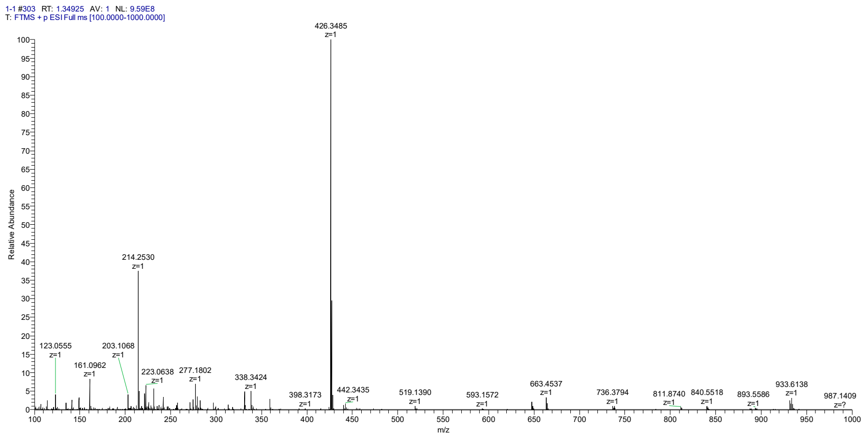

Figure S3. The mass spectrum of C_{14}PMimBr.

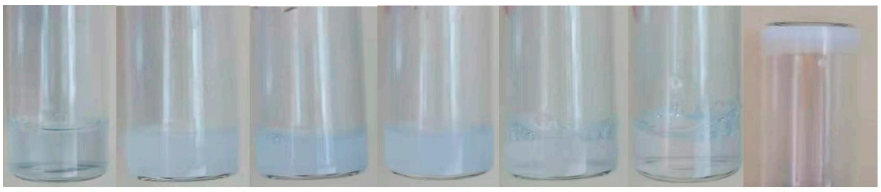

5mM **15mM** **20mM** **35mM** **50mM** **60mM** **75mM**

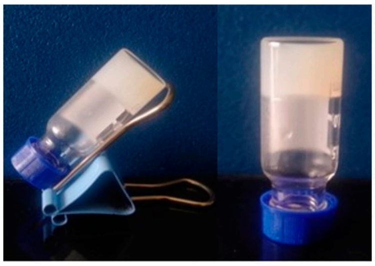

100mM

Figure S4. Optical photographs of C_{14}PMimBr (fixing molar ratio of BAMimBr-TDA

at 1:1) solutions with different concentration.

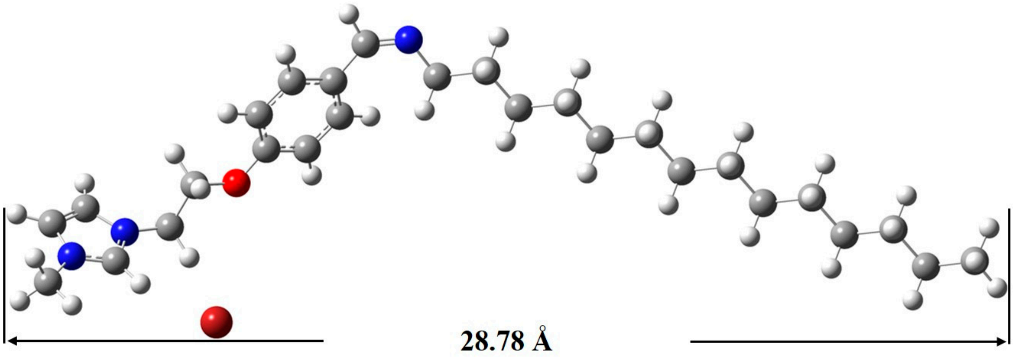

Figure S5. Geometries of C_{14}PMimBr molecules optimized using the polarizable continuum model at the B3LYP/6-31G(d,p) level.

The amount of adsorbed surfactant (Γ) at the air-water interface can be calculated using the Gibbs adsorption isotherm[1]:

$$\Gamma = -\frac{1}{nRT}\left(\frac{\partial \gamma}{\partial \ln C}\right)_T \text{ (mmol/m}^2\text{)} \qquad [1]$$

The minimum area occupied (A) by a surfactant molecule at the air-solution interface was obtained from the saturated adsorption as follows:

$$A_{\min} = \frac{1}{N_A \cdot \Gamma_{\max}} \quad (\times 10^{23}\text{Å}^2) \qquad [2]$$

The hydrocarbon chain length l can be calculated from the following Tanforde quation[2]:

$$l = 1.5 + 1.265N \qquad [3]$$

v_l is the volume of the hydrophobic part of the surfactant, calculated according to the Tanford equation:

$$v_l\,(\text{Å}^3) = 27.4 + 26.9N \qquad [4]$$

Table S1. The DFT calculation information of C_{14}PMimBr molecule.

Symbolic Z-matrix: Charge =0 Multiplicity = 1

C	-3.05691	1.28927	-1.05798
C	-2.23973	0.50304	-1.78513
C	-0.88758	1.74322	-0.49305
N	-2.26701	1.95731	-0.00533
H	-4.10249	1.41073	-1.22162

H	-2.53477	-0.20731	-2.52549
H	-0.02944	2.28151	-0.15248
C	-2.60816	3.37731	0.19043
H	-1.966	3.79497	0.93578
H	-2.47976	3.90746	-0.72775
H	-3.62566	3.46008	0.51032
N	-0.86818	0.81283	-1.39318
C	0.33734	0.18289	-1.94286
H	0.5811	-0.67485	-1.35104
H	0.1591	-0.11838	-2.95453
C	1.50891	1.18198	-1.89962
H	1.67457	1.49507	-0.89119
H	1.2782	2.03775	-2.50403
C	3.8186	1.40513	-2.29017
C	5.07195	0.85402	-2.00079
C	3.68698	2.79203	-2.4708
C	6.19265	1.67906	-1.89494
H	5.17143	-0.19987	-1.86048
C	4.81616	3.62166	-2.37505
H	2.7274	3.2172	-2.68114
C	6.07077	3.06008	-2.09102
H	7.14653	1.25345	-1.66451
H	4.72015	4.67981	-2.51529
O	2.68655	0.53911	-2.3948
C	8.62455	5.86458	-1.99425
H	9.39843	5.1525	-2.18485
H	8.69028	6.21473	-0.98421
C	8.80801	7.05738	-2.94932
H	8.0652	7.80708	-2.76542
H	8.72587	6.72987	-3.96697
C	10.22028	7.61674	-2.691
H	10.29133	7.94188	-1.67358
H	10.93172	6.83814	-2.87298
C	10.5391	8.8006	-3.61683
H	9.86273	9.61061	-3.42704
H	10.44772	8.49927	-4.63785
C	11.9885	9.23436	-3.3335
H	12.07967	9.53013	-2.30818
H	12.64124	8.40812	-3.52719
C	12.385	10.40736	-4.23849

H	11.75383	11.24706	-4.0379
H	12.28276	10.12357	-5.26523
C	13.85185	10.77118	-3.95198
H	14.47142	9.92336	-4.15968
H	13.95592	11.04793	-2.92418
C	14.28319	11.9469	-4.84069
H	13.67856	12.80035	-4.62236
H	14.16797	11.68183	-5.87104
C	15.76048	12.26915	-4.55713
H	16.35787	11.41241	-4.78888
H	15.87682	12.51991	-3.52451
C	16.2166	13.45627	-5.41754
H	15.63097	14.31794	-5.17228
H	16.09077	13.22463	-6.45427
C	17.70006	13.73745	-5.12809
H	18.28345	12.87992	-5.39516
H	17.82762	13.94378	-4.0858
C	18.15871	14.95437	-5.94553
H	17.57373	15.80533	-5.66791
H	18.02847	14.75587	-6.98947
C	19.64426	15.23874	-5.65958
H	20.23404	14.39517	-5.95465
H	19.77965	15.41919	-4.61303
C	20.08118	16.48332	-6.4553
H	19.49033	17.32434	-6.15461
H	21.11286	16.68714	-6.26008
H	19.94423	16.30776	-7.50049
N	7.3342	5.20301	-2.19303
C	7.34144	3.92598	-1.99123
H	8.27277	3.45479	-1.74771
Br	-0.33188	3.39558	-3.41272

Standard orientation:

Center Number	Atomic Number	Atomic Type	Coordinates (Angstroms) X	Y	Z
1	6	0	11.039234	-1.524428	0.531741
2	6	0	10.693141	-0.593889	-0.378750

3	6	0	9.107498	-0.502541	1.206569
4	7	0	10.191945	-1.361268	1.729149
5	1	0	11.798589	-2.260429	0.403512
6	1	0	11.210163	-0.369025	-1.285340
7	1	0	8.162741	-0.354424	1.683680
8	6	0	9.712480	-2.631253	2.302189
9	1	0	9.075708	-2.424322	3.135293
10	1	0	9.166241	-3.175266	1.563094
11	1	0	10.547772	-3.214391	2.628873
12	7	0	9.464717	0.041934	0.087555
13	6	0	8.743336	1.113226	-0.608284
14	1	0	9.115793	2.058146	-0.270944
15	1	0	8.894347	1.024606	-1.664387
16	6	0	7.239236	1.015209	-0.290964
17	1	0	7.091398	1.086557	0.765185
18	1	0	6.855217	0.076617	-0.640906
19	6	0	5.168409	2.089678	-0.597877
20	6	0	4.498424	3.311035	-0.464653
21	6	0	4.476182	0.884184	-0.395307
22	6	0	3.142550	3.334104	-0.134556
23	1	0	5.025362	4.227436	-0.615646
24	6	0	3.109231	0.906324	-0.073654
25	1	0	4.989133	-0.050987	-0.485643
26	6	0	2.443179	2.135460	0.051582
27	1	0	2.637710	4.270730	-0.025026
28	1	0	2.577110	-0.011402	0.078584
29	8	0	6.556503	2.092516	-0.937976
30	6	0	-1.183307	1.429953	0.915993
31	1	0	-1.449457	2.395075	0.541842
32	1	0	-1.269955	1.414748	1.983401
33	6	0	-2.148609	0.389009	0.321659
34	1	0	-1.929792	-0.589171	0.699689
35	1	0	-2.062376	0.375262	-0.746984
36	6	0	-3.570323	0.822928	0.727334
37	1	0	-3.646219	0.832082	1.795071
38	1	0	-3.747178	1.806098	0.343176
39	6	0	-4.636406	-0.126291	0.159197
40	1	0	-4.504372	-1.112149	0.559525
41	1	0	-4.557728	-0.166684	-0.905604
42	6	0	-6.014611	0.433281	0.554944

43	1	0	-6.089346	0.478880	1.622379
44	1	0	-6.118977	1.417370	0.146366
45	6	0	-7.134705	-0.457903	0.004192
46	1	0	-7.054317	-1.438579	0.423144
47	1	0	-7.057824	-0.519622	-1.061389
48	6	0	-8.487775	0.167408	0.384053
49	1	0	-8.555326	1.146189	-0.043990
50	1	0	-8.562059	0.235950	1.448753
51	6	0	-9.635192	-0.702192	-0.150168
52	1	0	-9.581255	-1.674423	0.289688
53	1	0	-9.558705	-0.786920	-1.214182
54	6	0	-10.974144	-0.038827	0.215596
55	1	0	-11.022500	0.928854	-0.238089
56	1	0	-11.043233	0.058296	1.277918
57	6	0	-12.143598	-0.896837	-0.288202
58	1	0	-12.105423	-1.858630	0.179880
59	1	0	-12.078050	-1.015370	-1.349308
60	6	0	-13.463823	-0.196345	0.072095
61	1	0	-13.505954	0.756107	-0.415850
62	1	0	-13.515822	-0.055702	1.131682
63	6	0	-14.645022	-1.066865	-0.382241
64	1	0	-14.596251	-2.012754	0.114066
65	1	0	-14.595452	-1.215567	-1.441300
66	6	0	-15.969279	-0.368434	-0.024440
67	1	0	-16.028874	0.570589	-0.535516
68	1	0	-16.012615	-0.201996	1.032247
69	6	0	-17.144652	-1.268479	-0.450437
70	1	0	-17.081875	-2.204891	0.065351
71	1	0	-18.068373	-0.789160	-0.203470
72	1	0	-17.102551	-1.439372	-1.504492
73	7	0	0.205259	1.159920	0.540274
74	6	0	0.941283	2.211824	0.387485
75	1	0	0.482260	3.174094	0.497146
76	35	0	7.268869	-2.120934	-1.152203

References

(1) Rosen, M. J.; Cohen, A. W.; Dahanayake, M.; Hua, X. Relationship of Structure to Properties in Surfactants. 10. Surface and Thermodynamic Properties of 2-Dodecyloxypoly(ethenoxyethanol)s, $C_{12}H_{25}(OC_2H_4)_xOH$, in aqueous solution. *J. Phys. Chem.* **1982**, *86*, 541-545.

(2) Tanford, C. Micelle Shape and Size. *J. Phys. Chem.* **1972**, *76*, 3020-3024.
